# Factors affecting the dental erosion severity of patients with eating disorders

**DOI:** 10.1186/1751-0759-8-25

**Published:** 2014-11-19

**Authors:** Mitsuhiro Otsu, Akira Hamura, Yuiko Ishikawa, Hiroyuki Karibe, Tomoyasu Ichijyo, Yoko Yoshinaga

**Affiliations:** Clinical Center of Psychosomatic Dentistry, Nippon Dental University Hospital, 2-3-16 Fujimi, Chiyoda-ku, Tokyo, 102-8158 Japan; Department of Pediatric Dentistry, School of Life Dentistry at Tokyo, Nippon Dental University, Tokyo, Japan; Department of Psychosomatic Internal Medicine, Kudanzaka Hospital, Tokyo, Japan; Hasegawa Hospital, Tokyo, Japan

**Keywords:** Eating disorders, Dentistry, Vomiting/PX, Vomiting/CO, Tooth erosion

## Abstract

**Background:**

Intraoral disease is a common occurrence in patients with eating disorders, particularly dental erosion, which frequently becomes severe and may hinder daily life. The severity varies from patient to patient. Understanding the underlying mechanisms may help prevent dental erosion in these patients. Accordingly, we investigated the relationship between the severity of erosion and the behavior of patients with eating disorders, with a focus on daily diet and vomiting behavior.

**Methods:**

A total 71 female eating disorder outpatients from the Clinical Center of Psychosomatic Dentistry of Nippon Dental University Hospital and the Psychosomatic Internal Medicine Department of Kudanzaka Hospital or who were hospitalized at Hasegawa Hospital were enrolled. Dental erosion severity and location were determined by oral examination. Patients who induced vomiting were queried on their behavior during vomiting and on routine diet habits. Patients with dental erosion were further divided into mild and severe groups based on the lesion severity and the groups compared.

**Results:**

Dental erosion was observed in 43 of 50 subjects who induced vomiting. Dental erosion was most frequent on the palatal side of the anterior maxillary teeth, occurring in 81.3% of the subjects. There were significant differences observed between the mild and severe groups according to post-vomiting oral hygiene. Significantly more subjects in the mild group consumed large amounts of water before vomiting, and significantly more subjects in the severe group routinely consumed carbonated beverages or sweetened food.

**Conclusions:**

While self-induced vomiting is the main cause of dental erosion in eating disorder patients, the erosion severity may be affected by behavior when inducing vomiting or by routine consumption of certain foods and beverages. Addressing these factors may help prevent severe dental erosion in patients who chronically induce vomiting.

## Background

Patients with eating disorders are more likely to demonstrate high levels of dental fear compared to other dental patients [1]. Because of the presence of dental fear, potential nutritional deficiencies, and exposure to stomach acids from frequent vomiting, eating disorder patients may have numerous dental caries and intraoral diseases such as salivary gland swelling and dental erosion [2, 3]. Dental erosion in particular is often observed in patients with eating disorders, with some patients requiring complex restorative treatment for this condition [4–7].

Generally, dental erosion is a disease that affects people who are directly or indirectly exposed to hydrochloric, sulfuric, and other acids through their occupations [8]. Recently, multiple studies have reported dental erosion due to overconsumption of carbonated beverages or citrus fruit [9, 10]. However, because these types of dental erosion usually progress gradually, patients usually do not experience symptoms such as pain induced by cold fluids, and even when symptoms do occur, the pain is minimal [9].

In contrast, dental erosion may progress quickly in eating disorder patients and interrupt daily life due to cold-induced pain or marked aesthetic damage [5, 11]. However, the severity of dental erosion varies among eating disorder patients and is not associated with the eating disorder’s duration [3, 5]. Instead, other factors are presumed be involved in the progression of dental erosion; determining these factors may help prevent severe damage.

Accordingly, we investigated the relation between the severity of dental erosion and the vomiting behavior and regular dietary habits of patients with eating disorders.

## Methods

### Subjects

A total of 71 female eating disorder patients (aged 17–47 years, mean 31.1 years), including outpatients at the Clinical Center of Psychosomatic Dentistry of Nippon Dental University Hospital, the Psychosomatic Internal Medicine Department of Kudanzaka Hospital, and inpatients at Hasegawa Hospital, were enrolled. A physician who specializes in psychosomatic medicine or a psychiatrist examined the subjects to exclude physical illnesses and other mental disorders. An eating disorder was diagnosed according to the criteria of the *Diagnostic and Statistical Manual of Mental Disorders*, 4th edition, text revision (DSM-IV-TR) [12]. The duration of the eating disorder ranged from 1 to 26 years (mean 11.7 years). Anorexia Nervosa (AN) was diagnosed in 35 patients (Restricting type = 11, Binge-Eating/Purging type = 24), Bulimia Nervosa (BN) in 27 (Purging type = 21, Nonpurging type = 6), Eating Disorder Not Otherwise Specified in 3, and unclear diagnosis in six patients. Patients with AN or BN who binge ate and purged through the use of laxatives only were not included in the study. Based on self-induced vomiting behavior, patients were further classified into vomiting (*n* = 50) and non-vomiting (*n* = 21) groups.

All patients received an oral and written study explanation, and provided written consent to participate. This study was approved by the ethics review committees of the School of Life Dentistry of Nippon Dental University, Nippon Dental University Hospital, Kudanzaka Hospital, and Hasegawa Hospital.

### Measurements

**Figure 1:**
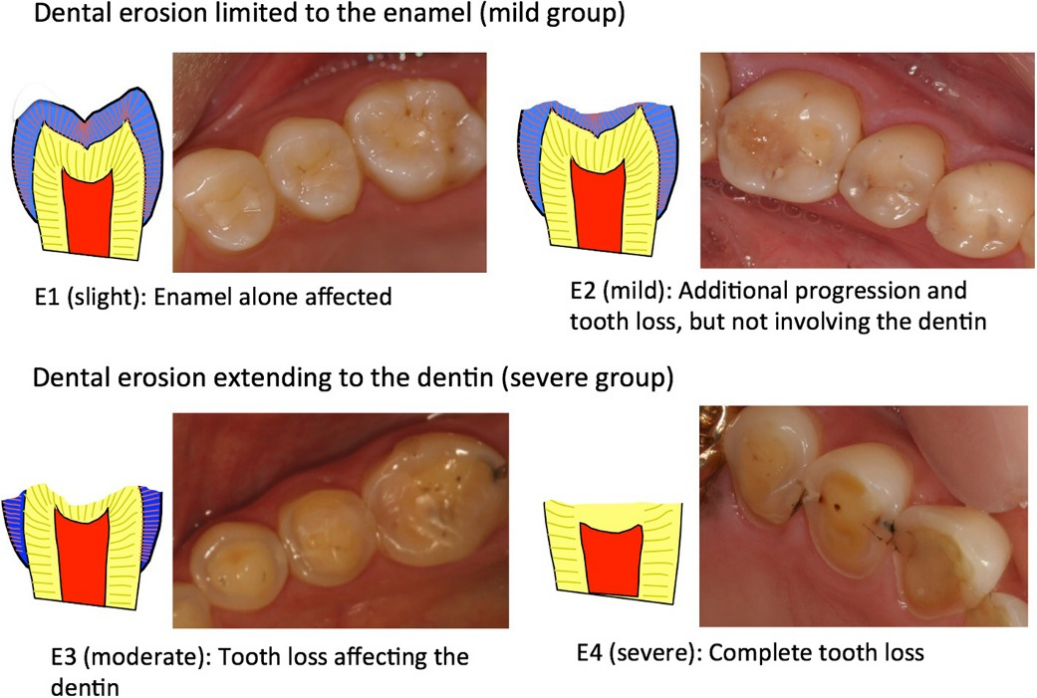
**Diagnostic criteria for dental erosion.** Dental erosion limited to the enamel (mild group). Dental erosion extending to the dentin (severe group). E1 (slight): Enamel alone affected. E2 (mild): Additional progression and tooth loss, but not involving the dentin. E3 (moderate): Tooth loss affecting the dentin. E4 (severe): Complete tooth loss.

The occurrence and severity of dental erosion was assessed by two dentists experienced in providing dental care to patients with eating disorders, using diagnostic criteria for dental erosion [8] and scored as E1 (slight) to E4 (severe) (Figure 1).

The 50 vomiting group subjects were assigned to one of two subgroups based on dental erosion severity. Subjects without dental erosion and those with erosion only involving the enamel (E1, E2) were placed into the mild group (*n* = 24). Subjects with erosion reaching the dentin with or without tooth loss (E3, E4) were placed in the severe group (*n* = 26).

Subjects were queried on their habits before and after vomiting through a standardized interview with the dentists (MO and YI). The interview included questions concerning water consumption to induce vomiting; post-vomiting oral hygiene; and regular dietary habits, such as consumption of acidic foods and beverages, citrus fruit, and sugar-sweetened foods such as candy or gum.

### Statistical analysis

Dental erosion frequency was compared between the vomiting and non-vomiting groups using the χ^2^ test. The mild and severe vomiting subgroups were compared using a *t*-test according to age and eating disorder duration, and a χ^2^ test for vomiting behavior and dietary habits.

## Results

### Self-induced vomiting and dental erosion frequency

**Figure 2:**
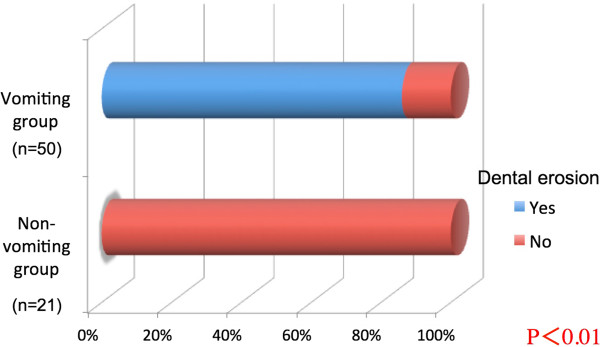
**Dental erosion frequency compared according to the presence of self-induced vomiting.**

Dental erosion was observed in 43 of 50 subjects (86%) in the vomiting group, and 0 of 21 subjects in the non-vomiting group, a difference that was statistically significant (P <0.01, Figure 2).

### Dental erosion severity and incidence by location

In the vomiting group, dental erosion occurred most frequently on the palatal side of the anterior maxillary teeth (81.3% of cases). Additionally, over half of the subjects exhibited erosion on the mandibular molar occlusal surface and the maxillary molar occlusal surface and palatal surface.

**Figure 3:**
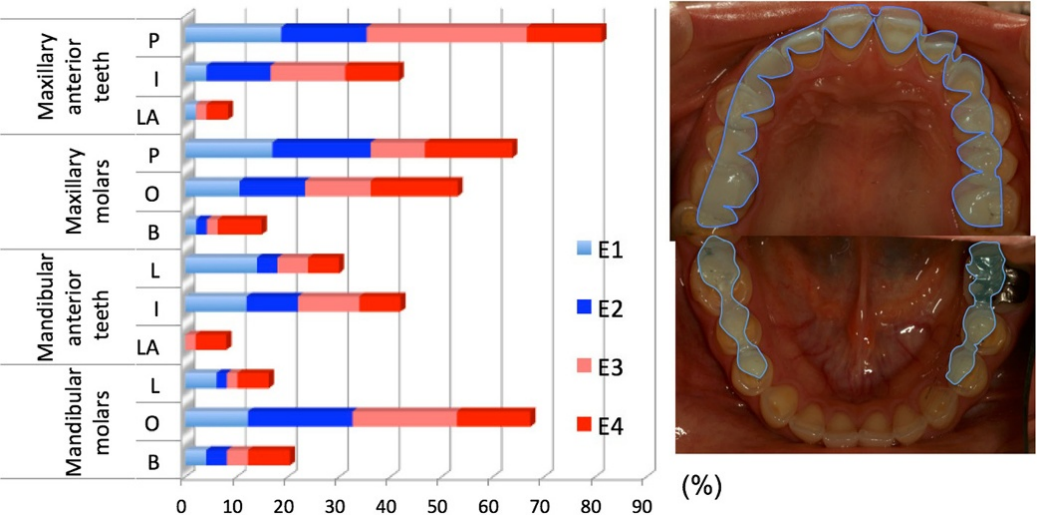
**Dental erosion severity and incidence by location: vomiting group.** P: palatal side; I: incisal side; LA: labial side; O: occlusal surface; B: buccal side; L: lingual side.

Mandibular molar occlusal surface erosion was scored as E3 or E4 grade severity in 34.7% of all subjects (Figure 3). Representative oral lesions are illustrated in Figure 3. Dental erosion was more frequent and severe in the maxillary teeth on the palatal side and in the mandibular molar occlusal surfaces.

### Dental erosion severity, patient age, and disease duration

The mean age and eating disorder duration for the mild and severe groups are shown in Table 1. There was no statistically significant difference between the groups.

**Table 1:** **Patient age and eating disorder duration**

	Mild group (n =24)	Severe group (n =26)	P value
Age (years)	31.7 ±7.2	30.0 ±7.1	0.43
Eating disorder duration (years)	11.2 ±6.5	8.7 ±6.3	0.67

Data represent the mean ± standard deviation.

Mild group, patients with dental erosion limited to the enamel; severe group, patients with dental erosion extending to the dentin.

### Dental erosion severity and behavior before and after vomiting

**Figure 4:**
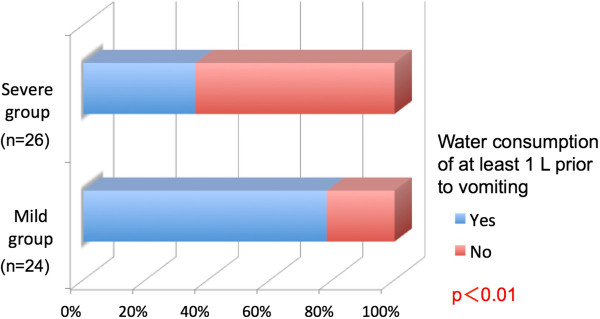
**Water consumption before vomiting compared according to the dental erosion severity.**

**Figure 5:**
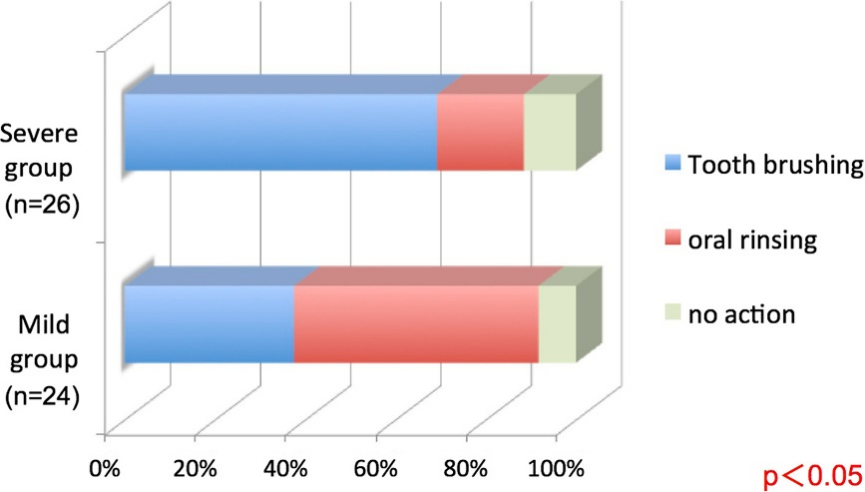
**Post-vomiting oral hygiene activities compared according to the dental erosion severity.**

Nineteen subjects (79.2%) in the mild group and nine subjects (34.6%) in the severe group consumed at least one liter of water to induce vomiting (χ^2^ test, P <0.01, Figure 4). Tooth brushing was performed by nine subjects (37.5%) in the mild group after vomiting, oral rinsing by 13 subjects (54.2%), and no action by two subjects (8.3%). In the severe group, tooth brushing was performed by 18 subjects (69.2%), oral rinsing by five subjects (19.2%), and no action by three subjects (11.5%). These differences were statistically significant (P <0.05, Figure 5).

### Dental erosion severity and routine dietary habits

**Figure 6:**
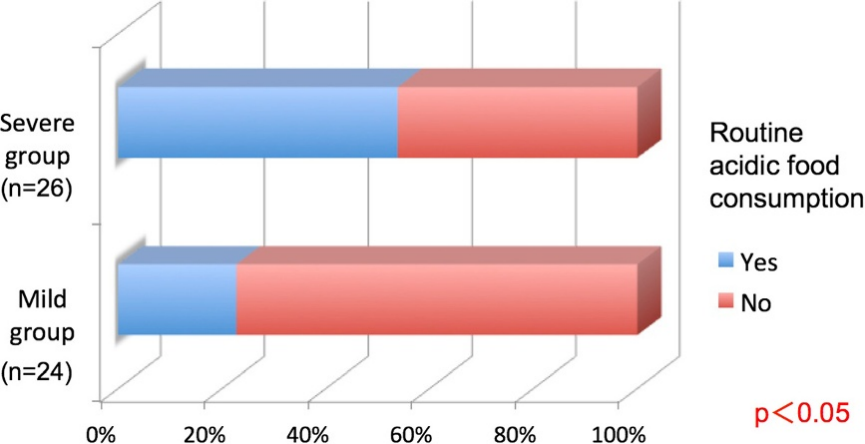
**Routine consumption of acidic foods compared according to the dental erosion severity.**

**Figure 7:**
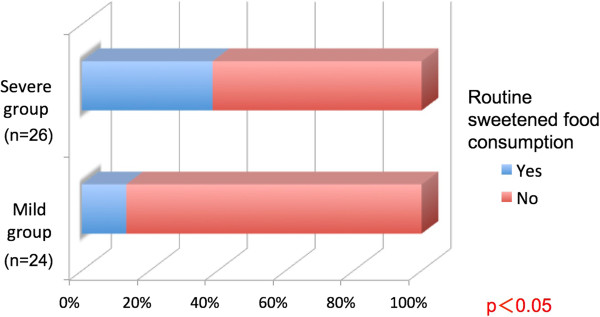
**Routine consumption of sweetened foods compared according to the dental erosion severity.**

Five subjects (20.8%) in the mild group and 14 subjects (53.8%) in the severe group regularly consumed acidic foods, such as carbonated beverages and citrus fruit (Figure 6). Three subjects (12.5%) in the mild group and ten subjects (38.5%) in the severe group regularly consumed sugar-sweetened foods such as candy and gum. Significant differences in both characteristics were observed between the groups (P <0.05, Figure 7).

## Discussion

Several epidemiological studies have investigated the prevalence of dental erosion. In the adult population, prevalence data ranged between 4% and 100% [13]. However, the methods and indices applied vary considerably, and this may limit the comparability of the prevalence data [14]. A recent study reported that dental erosion was observed in 8.8% of a healthy control group, and 45.0% of an eating disorder group [4]. In the present study, dental erosion was not observed in the non-vomiting group, while erosion was found in more than 80% of the vomiting group. This suggests that self-induced vomiting can cause dental erosion in patients with eating disorders. However, most notably, dental erosion was not observed in seven subjects in the vomiting group. This finding shows that vomiting is not always accompanied by dental erosion. Determining the protective mechanism underlying this finding could help preserve the teeth of patients with eating disorders, even if habitual self-induced vomiting cannot be stopped.

This study showed that dental erosion occurs most frequently on the palatal margin of the maxillary teeth as well as on the mandibular molar occlusal surfaces, where it is particularly severe. This is consistent with past studies characterizing endogenous dental erosion [15]. As vomitus passes through the oral cavity, dental erosion targets surfaces unprotected by the tongue, buccal mucosa, or lips—surfaces experiencing direct vomitus contact. In addition, the eroded dentin on occlusal surfaces may be further subject to damage during mastication. These characteristic lesions may help dentists recognize endogenous dental erosion and to consider the possibility of an eating disorder. Frequently, the dentist is the first health care provider to find the clinical symptoms of an eating disorder [16]. This may provide an opportunity to identify eating disorder patients who are not receiving medical attention. Thus, it is important for dentists to accurately recognize the pattern of dental erosion associated with eating disorders.

Dental erosion was less common in areas protected from direct acid exposure by the tongue and other structures. Using a device, such as a mouth guard, during vomiting could similarly suppress dental erosion [11]. However, this intervention could also be interpreted as condoning the act of vomiting and warrants full debate.

Dental erosion severity did not correlate with the eating disorder duration or patient age. However, it is generally thought that acid exposure for longer periods and with more frequency increases the severity of tooth erosion. The subjects were not followed long-term but only underwent a one-time cross-sectional survey; therefore, the relationship between erosion severity and vomiting frequency and disease duration requires further study.

A large proportion of subjects in the mild group consumed water before inducing vomiting. Drinking water immediately before vomiting is thought to dilute the gastric contents and neutralize the vomitus acidity, thereby reducing dental erosion severity. In addition, diluted vomitus flows more easily, which may decrease residual vomitus in the mouth. The impact of significant pre-vomiting water consumption on the stomach, esophagus, and other organs warrants investigation from an internal medicine perspective. Our results suggest that this behavior can effectively protect the teeth from acid.

This study also showed that tooth brushing immediately after vomiting is harmful, a finding consistent with past studies showing that brushing after vomiting or eating acidic foods etches the acid onto tooth surfaces [17]. In addition, brushing immediately after vomiting can remove the superficial tooth surface down to the decalcified layer, which can remineralize. A recent study has shown that approximately 83% of subjects who brushed their teeth following vomiting episodes suffered from dental erosion compared with subjects who only rinsed their mouth or did nothing [18]. Therefore, to avoid worsening dental erosion, post-vomiting oral hygiene should comprise thorough oral rinsing with water or other liquids to neutralize acid in the oral cavity [19], waiting to allow remineralization, and careful tooth brushing.

Excessive consumption of acidic foods is believed to promote dental erosion [20, 21]. The critical pH of surface enamel is approximately 5.5, and approximately 6.7 in the exposed dentin. Generally, carbonated beverages and citrus fruit have a pH of approximately 3.0, more than capable of dissolving the tooth. Therefore, regular consumption of these products may cause chronic oral acidity, which may exacerbate dental erosion caused by vomiting. In addition, oral bacteria including *Streptococcus mutans* metabolize consumed sugars and produce lactic acid and other acids, generating oral acidity and promoting dental erosion. However, even in oral acidity caused by consuming acidic or sweetened foods, the teeth will remineralize after a certain period. Thus, if the oral cavity pH can be neutralized before tooth remineralization begins, dental erosion can be minimized, even if these food products are consumed.

For eating disorder patients, sugar consumption is often an important source of calories, and prohibiting these patients from consuming sweetened foods risks morbidity or fatality [22]. As such, the progression of dental erosion should be slowed by changing how patients consume sweetened foods or by modifying behaviors after consumption, such as avoiding continuous consumption or rinsing the mouth afterwards.

## Conclusions

Self-induced vomiting is the main cause of dental erosion in patients with eating disorders. Erosion occurs most frequently on the palatal side of the maxillary teeth and the mandibular molar occlusal surfaces, which come into direct contact with vomitus and gastric acid. Many patients exhibit severe erosion of the molars. Disease duration and patient age did not correlate with erosion severity.

This study also revealed that water consumption prior to vomiting and oral rinsing afterwards may help prevent erosion from progressing from mild to severe. Conversely, tooth brushing after vomiting had a detrimental effect. Not surprisingly, regular consumption of acidic or sweetened foods was associated with progression to severe erosion. Addressing these factors could help prevent the progression of dental erosion in patients who chronically induce vomiting.
